# High PD‐L1 expression in the tumour cells did not correlate with poor prognosis of patients suffering for oral squamous cells carcinoma: A meta‐analysis of the literature

**DOI:** 10.1111/cpr.12537

**Published:** 2018-11-15

**Authors:** Giuseppe Troiano, Vito C. A. Caponio, Khrystyna Zhurakivska, Claudia Arena, Giuseppe Pannone, Marco Mascitti, Andrea Santarelli, Lorenzo Lo Muzio

**Affiliations:** ^1^ Department of Clinical and Experimental Medicine University of Foggia Foggia Italy; ^2^ Department of Clinical Specialistic and Dental Sciences Marche Polytechnic University Ancona Italy; ^3^ Dentistry Clinic National Institute of Health and Science of Aging, INRCA Ancona Italy

**Keywords:** cancer, checkpoint inhibitors, immunity, meta‐analysis, mouth neoplasms, PD‐1, PD‐L1

## Abstract

**Objectives:**

Oral cancer represents one of the most common malignancies in humans. Its prognosis is still poor, despite the most recent improvements in therapies. An increasing attention is placed on the role of programmed death ligand 1 (PD‐L1) in the tumour immunity and its potential function as a marker for tumour prognosis. Whether PD‐L1 expression is a prognostic factor for the poor outcomes in oral squamous cell carcinoma is still controversial. This study aimed to investigate, through a meta‐analysis, a potential correlation between PD‐L1 expression and the prognostic outcomes in patients with oral squamous cell carcinoma.

**Materials and methods:**

The studies were identified by searching PubMed, SCOPUS, Web of Science and were assessed by two of the authors. After the selection process, 11 articles met eligibility criteria and were included in the meta‐analysis. Quality assessment of studies was performed according to the REMARK guidelines, and the risk of biases across studies was investigated through Q and *I* ^2^ tests. Meta‐analysis was performed to investigate the association between the PD‐L1 expression either overall survival (OS), disease‐free survival (DFS), disease‐specific survival (DSS), gender and lymph node metastasis.

**Results:**

A total of 1060 patients were analysed in the 11 studies included in the meta‐analysis. Pooled analysis revealed that the expression of PD‐L1 did not correlate with poor OS (HR, 0.60; 95% CI: [0.33, 1.10]; *P* = 0.10), DFS (HR, 0.62; 95% CI: [0.21, 1.88]; *P* = 0.40), DSS (HR, 2.05; 95% CI: [0.53, 7.86]; *P* = 0.29 and lymph node metastasis (HR, 1.15; 95% CI: [0.74, 1.81]; *P* = 0.53). Furthermore, results of the meta‐analysis showed that high expression of PD‐L1 is two times more frequent in female patients (OR, 0.5; 95% CI: [0.36, 0.69]; *P *< 0.0001) compared to males. For all the three outcomes analysed, a high rate of heterogeneity was detected (*I* ^2^ *> *50%).

**Discussion:**

High PD‐L1 expression did not correlate with poor prognosis of patients suffering for oral squamous cell carcinoma. Studies published on the topic showed a significant variation in results, limiting the use of PD‐L1 expression by immunohistochemistry as prognostic biomarker in clinical practice.

## 1 INTRODUCTION

Oral squamous cells carcinoma (OSCC) represents one of the most common malignancies in humans.^1^ An annual incidence of about 200 000 new cases per year has been estimated worldwide.^2^ Both incidence and mortality rate are about 2.8 times higher in males than in females.^3^ The most known risk factors for the onset of OSCC are tobacco smoke, betel chew and alcohol consumption.^4^ The prognosis of OSCC is still poor, showing very little improvements in the last decades, despite advances in therapies.^5^ Recently, immunotherapy showed promising effects for the treatment of such patients.^6^ The results of several studies suggest an important role of immune evasion mechanisms in the pathogenesis of OSCC. For these reasons, a deeper understanding of molecules involved in the function of immune system is crucial for the development of future strategies of treatment.

As it is known, cancer cells can negatively regulate the immune response through the activation of inhibitory immune checkpoints. To date, different inhibitory immune checkpoints have been studied, including cytotoxic T‐lymphocyte protein 4 (CTLA4), programmed cell death protein 1 (PD‐1), lymphocyte activation gene‐3 (LAG3), T‐cell immunoglobulin‐3 (TIM3) and T‐cell immunoglobulin and ITIM domain (TIGIT).^7^ In this article, we focused on the PD‐1 immune checkpoint as the pharmacological inhibition of this immune checkpoints has recently demonstrated to improve the survival rate of patients with head and neck squamous cells carcinoma (HNSCC),^8^ while the power of evidence is still weak regarding the clinical efficacy of the pharmacological inhibition of the other immune checkpoints above mentioned. In particular, we reviewed studies focused on the analysis of the programmed cell death ligand‐1 (PD‐L1) as a prognostic factor of patients suffering for OSCC. PD‐L1 is a cell surface glycoprotein which induces both anergy and apoptosis of T cells through the activation of PD‐1 receptors located on their surface.^9^ The biological importance of the PD‐1 receptors influences significantly the immune responses because of a diffused ligand distribution in the body. In fact, such axis showed to play a crucial role in autoimmunity,^10^ tumour immunity,^11^ infectious immunity^12^ and allergy.^13^ PD‐L1 is commonly expressed in some healthy tissues since it is involved in the normal immunological homeostasis.^14^ However, in many types of cancer, the expression of PD‐L1 on tumour cells is remarkably higher. This overexpression seems to be present also in subsets of immune cells, including B and T cells, macrophages and dendritic cells.^11^ Several studies demonstrated a strong correlation between PD‐L1 expression on various tumour cells and a worse patients' prognosis.^15^, ^16^, ^17^, ^18^ Many studies have also been conducted to discover a possible role of the PD‐1/PD‐L1 axis in the biology of OSCC.^19^, ^20^ Its potential clinical and pathological implication has also been investigated providing, however, non‐homogeneous conclusions.

The aim of the present study was to systematically review the literature and perform a meta‐analysis on the available data in order to summarize the possible correlations between PD‐L1 expression and the prognosis of patients suffering for OSCC.

## 2 MATERIALS AND METHODS

### 2.1 Protocol and Registration

This systematic review has been carried out following the guidelines of the “Preferred Reporting Items for Systematic Reviews and Meta‐Analyses” (PRISMA) guidelines^21^ and the Cochrane Handbook.^22^ In addition, the protocol for the development of this review was prospectively registered on the online database PROSPERO (International prospective register of systematic reviews) with the registration number CRD42018090716.

### 2.2 Eligibility criteria

The inclusion criteria were the following: (a) both prospective and retrospective clinical cohort studies, written in English language, regarding the immunohistochemical evaluation of PD‐L1 expression in samples from OSCC patients; (b) at least 20 patients were included in each study; (c) studies which analysed the prognosis calculating the hazard ratio (HR) and its 95% confidence interval (95% CI) for at least one of the following: overall survival (OS), disease‐free survival (DFS), disease‐specific survival (DSS), gender and lymph node metastasis. Some studies reported the HR and 95% CI in the article. Others only reported the Kaplan‐Meier graph. In this case, the HR and 95% CI were extracted by Kaplan‐Meier graph using the method reported by Tierney et al.^22^ If the article did not report both HR and 95% CI, or the Kaplan‐Meier graph, author was contacted by email. By this last method, we got the HR and 95% CI for two studies.^23^, ^24^ Studies on non‐human model, case series with less than 20 patients and case reports were not considered for the inclusion in this review. No restrictions were applied about the year of publication.

### 2.3 Information sources and search strategy

Two authors (GT and KZ) performed an independent direct online search on the following databases: PUBMED, SCOPUS and Web of Science. The research process was carried out by two reviewers in an independent manner. MeSH terms and free text words were combined using Boolean operators (AND, OR). The following protocol was used: ((((PD‐L1 OR Programmed Death Ligand 1 OR checkpoint inhibitor OR immune system))) AND ((OSCC OR "oral cancer" OR Tongue OR gingiva))) AND ((survival OR prognosis OR biomarker)).

### 2.4 Study selection, data collection process and data items

The selection process was performed in two rounds. In the first round, authors screened the studies reading only title and abstract of publications, while in the second phase, a full‐text evaluation was performed. In case of disagreement between reviewers, a final decision for the inclusion was taken in a joint session with a third author (VCAC). This author also calculated a value of k‐statistic to show the level of reviewers' agreement. At the end of the selection process, papers fulfilling all inclusion criteria were included in the quantitative synthesis. Data extraction was performed using an ad hoc extraction sheet by two authors (VCAC and CA) in a joint session and controlled by a third author (GT). For each study, the following data were extracted: name of the first author, year of publication, name of the country where the study was performed, classification used for staging, number of patients included, cut‐off values, gender, staging, tumour size, rate of lymph node metastasis, HRs and 95% CI for the survival outcomes considered.

### 2.5 Risk of bias assessment

The risk of bias of the included studies was evaluated using a classification derived from the Reporting Recommendations for Tumour Marker Prognostic Studies (REMARK),^25^ as previously reported by Almangush et al.^26^ The scale consists of six parameters evaluating (a) samples, (b) clinical data of the cohort, (c) immunohistochemistry, (d) prognosis, (e) statistics and (f) classical prognostic factors. In addition, each parameter was considered as adequate, inadequate or not evaluable on the basis of the REMARKS guidelines. In addition, analysis of the risk of biases across studies was investigated through Q and *I* ^2^ tests. A *P*‐value of Q‐statistic <0.05 was considered significant for the presence of heterogeneity. The Higgins index was also assessed and classified as follows: low heterogeneity (<30%), medium heterogeneity (30%‐60%) and high heterogeneity (>60%).^27^

### 2.6 Summary measures and planned methods for analyses

For the pooled analysis of PD‐L1 expression as prognostic factor in OSCC patients, the natural logarithm of HR and its standard error (SE) were calculated and entered into the software: Review Manager version 5.2.8 (Cochrane Collaboration, Copenhagen, Denmark; 2014). The inverse of variance test was used to calculate the overall effect. Results of the meta‐analysis were summarized in forest plots, and a *P*‐value lower than 0.05 was considered as threshold of statistical significance for all the tests performed in this meta‐analysis. Sensitivity analyses were performed for the outcomes OS and DFS omitting articles on the basis of risk of bias, cut‐off and geography, hence repeating meta‐analysis through a random effect model.

## 3 RESULTS

### 3.1 Study selection

**Figure 1:**
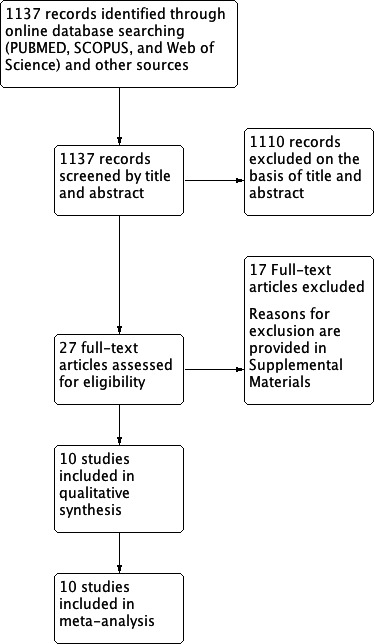
Flowchart for inclusion of studies in the meta‐analysis

A total of 1137 records were screened by title and abstract. Of these, only 27 overcame the first selection process and were included in the full‐text evaluation. Among these, only 10 studies met the inclusion criteria and were included in the meta‐analysis.^19^, ^23^, ^24^, ^28^, ^29^, ^30^, ^31^, ^32^, ^33^, ^34^ The flow chart of the selection process is reported in Figure 1, while reasons for exclusion of the remaining 17 articles are provided in Table S2.^33^, ^35^, ^36^, ^37^, ^38^, ^39^, ^40^, ^41^, ^42^, ^43^, ^44^, ^45^, ^46^, ^47^, ^48^, ^49^, ^50^ The value of *k*‐statistic was 0.8196 revealing an excellent level of agreement between reviewers (major details are available in Table S1).

### 3.2 Study features and risk of bias within studies

A total of 1060 patients were analysed in the 10 studies included in the meta‐analysis.^19^, ^24^, ^28^, ^29^, ^30^, ^31^, ^32^ Five studies were performed in Asia,^19^, ^28^, ^29^, ^30^, ^33^ two in Europe,^32^, ^34^ while the remaining three in other parts of the world (Brazil,^24^ Australia^31^ and USA^23^). The year of publication ranged from 2011 to 2018. Multivariate analysis was performed in two studies,^24^, ^29^ while the remaining eight^19^, ^28^, ^30^, ^31^, ^32^, ^33^, ^34^, ^51^ reported only results for univariate analysis. Three studies fully respected the REMARKS guidelines,^19^, ^28^, ^29^ while the remaining seven proved to be lacking in some of the parameters analysed.^23^, ^24^, ^30^, ^31^, ^32^, ^33^, ^34^, ^51^ Absence of risk of bias was detected only for the immunohistochemistry, while some deficiencies were present for the others parameters. Results of the risk of bias for each of the included study are reported in Table 1.

**Table 1:** Evaluation criteria used to assess the quality of studies included in the meta‐analysis according to the REMARK guidelines are reported in the Almangush et  al^25^ article.—Included Studies were evaluated as A: Adequate; I: Inadequate; N/A: no description

Author (year)	Country	Samples	Clinical data	Immunohistochemistry	Prognostication	Statistics	Classical Prognostic Factors
Ahn (2016)	Korea	A	A	A	A	A	A
Cho (2011)	Korea	A	A	A	A	A	A
Kogashiwa (2017)	Japan	A	A	A	A	A	A
Lin (2015)	Taiwan	I	A	A	A	A	A
Oliveira‐Costa (2015)	Brazil	I	A	A	I	I	A
Satgunaseelan (2016)	Australia	A	A	A	I	I	I
Straub (2016)	Germany	A	A	A	I	I	I
Hirai (2016)	Japan	I	A	A	I	A	A
Troeltzsch (2016)	Germany	A	A	A	I	A	A
Mattox (2017)	USA	I	I	A	I	I	I

### 3.3 Synthesis of results and risk of bias across studies

Meta‐analysis of seven studies revealed no significant correlation between high/low expression of PD‐L1 and OS (HR, 0.60; 95% CI: [0.33, 1.10]; *P* = 0.10). A high rate of heterogeneity was detected (*I* ^2 ^= 89%), and for such reason, a random effects model was used. Meta‐analysis of studies for DFS revealed no statistical significant differences between the expression of PD‐L1 in the tumour cells and DFS (HR, 0.62; 95% CI: [0.21, 1.88]; *P* = 0.40). Also for DFS, results obtained on the analysis of three studies showed a high rate of heterogeneity (I^2 ^= 81%). No significant differences were also detected for the rate of lymph node metastasis (HR, 1.15; 95% CI: [0.74, 1.81]; *P* = 0.53). On the basis of the extracted data, meta‐analysis was also performed for the secondary outcomes: gender and tumour size. Results for DSS (Figure S1) revealed the absence of a statistical difference between the high and low expression of PD‐L1 (HR, 2.05; 95% CI: [0.53, 7.86]; *P* = 0.29).The cumulative Odds Ratio (OR) for gender status showed that high expression of PD‐L1 is two times more frequent in female patients (OR, 0.5; 95% CI: [0.36, 0.69]; *P* < 0.0001). The rate of heterogeneity was *I* ^2 ^= 0%, and for such reason, a fixed effects model was used. Summary effect size for OS did not substantially change in sensitivity analyses performed including only studies at low risk of bias (HR = 0.55 [0.24, 1.28] *P *= 0.17), with an equal cut‐off (intensity > 2) (HR = 0.73 [0.27, 1.98] *P = *0.54) and performed only in Asia (HR = 0.55 [0.24, 1.28] *P = *0.17) (Figure S2). Sensitivity analysis was not performed for DFS and DSS because of the little number of studies included. Characteristics of included studies and their relative results are summarized in Tables 2, 3 and 4.

**Table 2:** Main characteristics of included studies

Study	Year	Country	No of patients	Staging edition	Detection method	Cut‐off
Ahn H.	2017	South Korea	68	7th AJCC	IHC	Intensity >2
Cho Y‐A.	2011	South Korea	45	7th AJCC	IHC	Score >2
Kogashiwa Y.	2017	Japan	84	N/A	IHC	>5% of tumour cells
Lin Y‐M.	2015	Taiwan	305	7th AJCC	IHC	Score >2
Oliveira‐Costa J. P.	2015	Brazil	96	N/A	IHC	>5% of tumour cells
Satgunaseelan L.	2016	Australia	217	7th AJCC	IHC	>5% of tumour cells
Straub M.	2016	Germany	80	7th AJCC	IHC	>5% of tumour cells
Mattox A. K.	2017	USA	53	N/A	IHC	>1% of membranous PD‐L1 expression by tumour and/or immune cells
Hirai M.	2016	Japan	24	N/A	IHC	>10% of tumour cells
Troeltzsch M.	2016	Germany	88	7th AJCC	IHC	Score >2

N/A: not reported.

**Table 3:** Synthesis of data extracted from the included studies related to outcomes pooled in the meta‐analysis

Study	Follow‐up	Overall survival		Disease‐free survival		HR estimation
		HR	95% CI	HR	95%CI	
Ahn H.	44.3 mean (2.1 to 122 months)	0.32	0.11‐0.94	0.25	0.06‐1.12	Reported
Cho Y‐A.	over 125 months/not reported	1.10	N/A	N/A	N/A	Calculated
Kogashiwa Y.	40.6 mean (3.8 to 89.6 months)	0.256	0.101‐0.646	N/A	N/A	Reported
Lin Y‐M.	45,6 mean (1,2 to 133,2 months)	1.209	0.890‐1.643	N/A	N/A	Reported
Oliveira‐Costa J. P.	20 mean (4 to 108 months)	0.426	0.186‐0.977	N/A	N/A	Reported
Satgunaseelan L.	22 median (1 to 144 months)	N/A	N/A	1.46	N/A	Calculated
Straub M.	31 mean (2 to 63 months)	N/A	N/A	2.11	1.00‐4.43	Calculated
Mattox A. K.	N/A	1.622	0.5‐4.464	N/A	N/A	Reported
Hirai M.	N/A	N/A	N/A	N/A	N/A	N/A
Troeltzsch M.	N/A	N/A	N/A	N/A	N/A	N/A

N/A: not reported.

**Table 4:** PD‐L1 expression in Lymph node metastasis (LNM) and Gender Status patients

PD‐L1 Expression									
Study	Country	High with LNM	Low with LNM	High in male	Low in male	High in female	Low in female	High expression	Low expression
Ahn H.	South Korea	N/A	N/A	N/A	N/A	N/A	N/A	45	23
Cho Y‐A.	South Korea	8	8	18	14	8	5	26	19
Kogashiwa Y.	Japan	31	29	24	33	20	7	44	40
Lin Y‐M.	Taiwan	52	64	93	143	40	29	133	172
Oliveira‐Costa J. P.	Brazil	Unclear	Unclear	Unclear	Unclear	Unclear	Unclear	Unclear	Unclear
Satgunaseelan L.	Australia	18	76	17	113	23	64	40	177
Straub M.	Germany	26	19	23	31	13	13	36	44
Hirai M.	Japan	1	4	N/A	N/A	N/A	N/A	13	11
Troeltzsch M.	Germany	18	27	13	35	13	27	26	62
Mattox A. K.	USA	N/A	N/A	N/A	N/A	N/A	N/A	N/A	N/A

N/A: not reported; unclear: data were reported but they were not clear.

## 4 DISCUSSION

PD‐L1, also known as B7‐H1 or CD274, is a cell surface glycoprotein, which leads to T‐cell inactivity or apoptosis by binding PD‐1, a receptor expressed by the T lymphocytes.^19^ The interaction between PD‐1/PD‐L1 leads to immune system impairment through a range of mechanisms, which often differs between tumour types. Once PD‐1 binds to PD‐L1, an inhibitory signal is induced. This happens through the phosphorylation of the tyrosine residue in the immunoreceptor tyrosine‐based switch motif, leading to the recruitment of SH2‐domain containing tyrosine phosphatase 2 (SHP‐2) to the cytoplasmic domain of PD‐1, which then down‐regulates CD28‐mediated PI3K activity. These events, ultimately, lead to reduction of Akt activation, which is involved in the proliferation and cytokine production from the immunity cells.^52^, ^53^ PD‐1 activation is also linked to inhibition of the anti‐apoptotic protein Bcl‐xL.^54^ In OSCC, many studies showed different links between PD‐1/PD‐L1 pathway and other molecules. Chen et al reported in an in vitro study that IFN‐γ causes an increase of PD‐L1 expression on the surface of the OSCC cell line, through PKD2 signalling pathway.^36^ However, this seems to contradict the description of the inhibitory effect of INF‐γ on cancer proliferation, showing an opposite role as cancer immune resistance.^55^ Ahn et al performed an immunohistochemical study on OSCC samples demonstrating that miR‐197 expression is inversely correlated with PD‐L1 expression. This relation had been already shown in non‐small cell lung cancer (NSCLC), where miR‐197 blocks the cyclin‐dependent kinase CKS1B, which is linked to PD‐L1 expression through STAT3 signal.^28^ Jingjing et al^56^ reported that protein level of PD‐L1 in OSCC cell line is higher than normal oral mucosa cell line, while no differences were highlighted in the PD‐L1 mRNA. They justified these statements by showing that ubiquitination could be the main mechanism involved in the PD‐L1 expression in OSCC cell lines, targeting USP9X as the main molecule acting as deubiquitinase, and this mechanism leads to the PD‐L1 protein accumulation.

The literature is still lacking studies regarding action and role of PD‐1/PD‐L1 pathway in OSCC cells. Recently, there has been growing interest about the PD‐L1 expression in tumour‐associated macrophages (TAM) and fibroblasts. Next studies should integrate findings coming from both tumour and peritumoral microenvironment PD‐L1 expression to improve the understanding of its role in OSCC prognosis.

Different studies showed that tumour cells could express on their surface PD‐L1, suggesting a potential role of this protein in reducing the anti‐cancer immune response.^19^ These findings improved the research in anti‐cancer drug development, which could interact with the PD‐1/PD‐L1 pathway.

On November 2016, the FDA approved a new pharmacological principle, nivolumab for the treatment of recurrent or metastatic head and neck squamous cell carcinoma. Nivolumab stands for a human IgG4 PD‐1 immune checkpoint inhibitor antibody, which selectively counters the link between PD‐1 and its ligand (PD‐L1), promoting the action of T‐cell function.^57^ Although the promising role of these new drugs, there are still problems about their controversial activity, above all the different mechanisms, in which PD‐1/PD‐L1 could also be involved in different cancer types. For example, for the NSCLC, not all tumours expressing PD‐L1 respond to PD‐1/PD‐L1 inhibitors. Conversely, some PD‐L1‐negative tumours can respond to these agents.^58^ However, the predictive role of PD‐L1 expression in tumour samples is still controversial.^59^

**Figure 2:**
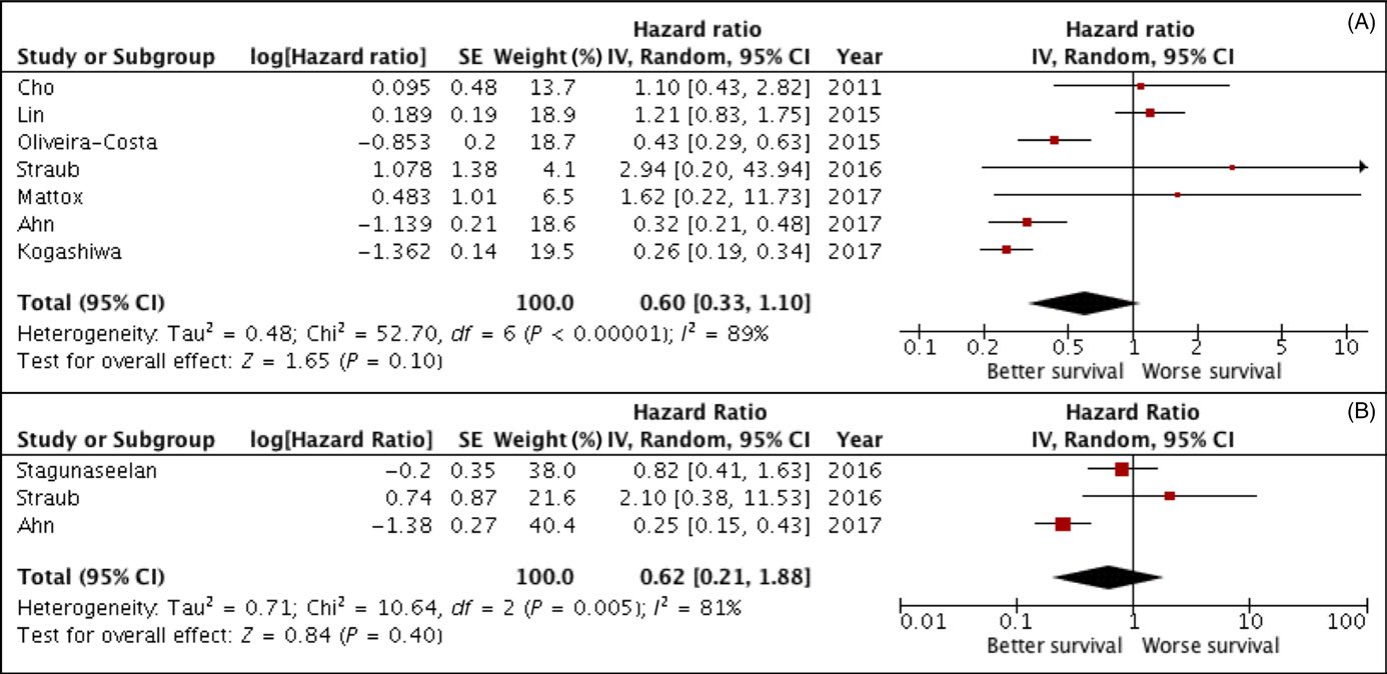
(A/B) Forest plot for the association of higher PD‐L1 expression with overall survival (A) and disease‐free survival (B)

**Figure 3:**
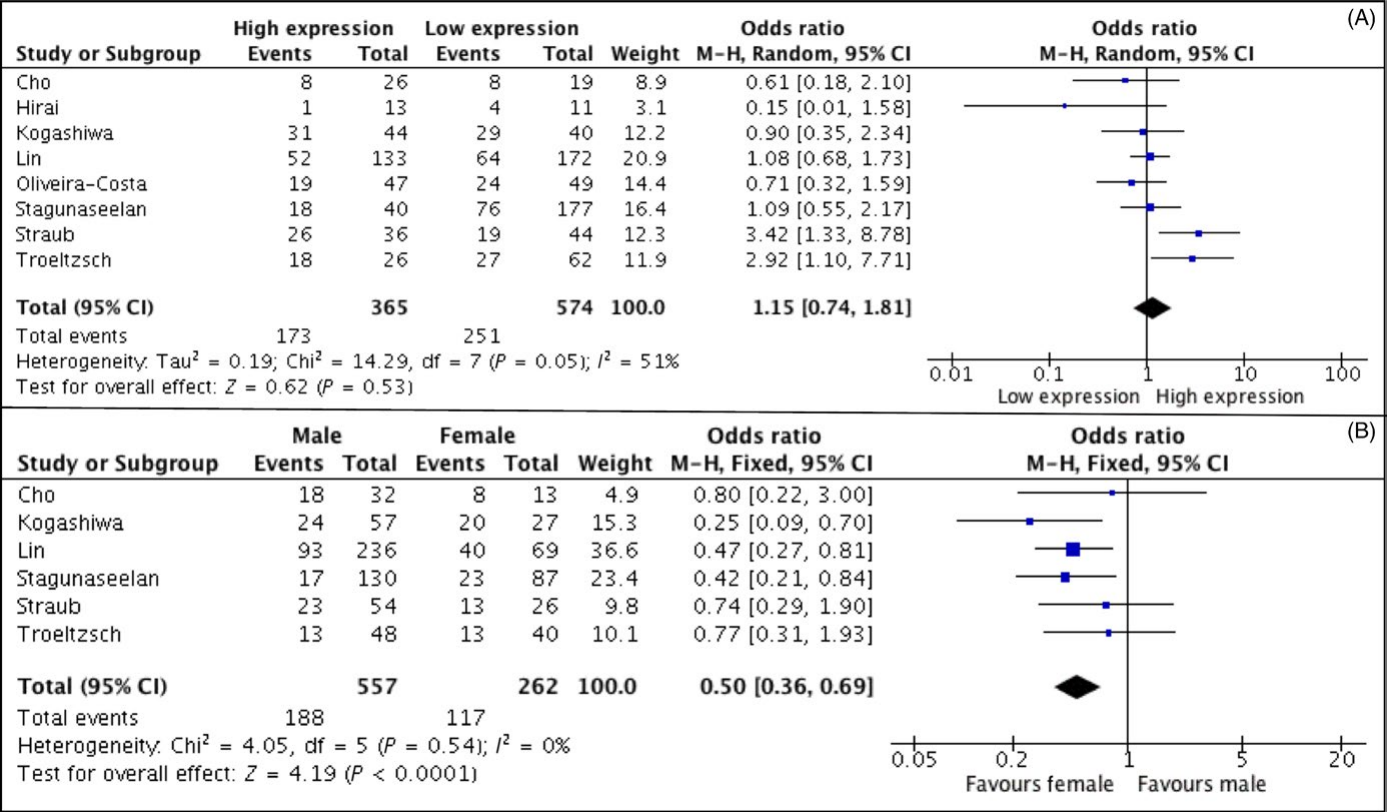
(A/B) Forest plot showing the association of higher PD‐L1 expression with lymph node metastasis (A) and gender status (B)

In this study, we focused on the analysis of PD‐L1 expression in OSCC tissue as a prognostic (and not predictive) biomarker. In fact, such marker has demonstrated to be an independent prognostic factor in different cancer types, including NSCLC,^60^ renal cell carcinoma^61^ and breast cancer.^62^ However, there are conflicting evidences in relation to the prognostic value of PD‐L1 in different types of cancer.^63^, ^64^, ^65^ Results of this study failed to reveal a correlation between the expression of PD‐L1 in tissues and a poor prognostic of OSCC patients. For both OS and DFS, the rate of heterogeneity among studies resulted to be very high, demonstrating that the results of the included studies are strongly conflicting among each other (Figures 1 and 2). No differences were also detected for the rate of lymph node metastasis in patients with higher PD‐L1 expression (Figure 3). Our findings are in discordance with the results of a previous meta‐analysis on head and neck cancers in which authors revealed a significant association between PD‐L1 expression and poor prognosis in a subgroup analysis.^66^ Such discrepancy is in part due to the inclusion of the meta‐analysis of two recently published studies in which PD‐L1 expression correlated with a better prognosis.^28^, ^29^

The lack of correlation between PD‐L1 expression and OS appears to contrast with the prognostic value that is attributed to this marker, based on its immunosuppressive function. Several studies regarding other tumour types found the same results, suggesting a more complex function of PD‐L1 in immunosurveillance signalling.^67^ A possible explanation is that PD‐L1 expression by cancer cells can be considered as a marker of an active host anti‐tumour immune response.^68^ Another way to address the issue is to consider the heterogeneity of tumour microenvironment in different tumour types. In fact, a classification of tumours into 4 types based on the presence of PD‐L1 positivity and/or tumour‐infiltrating lymphocytes has been proposed.^68^, ^69^ In some tumours like NSCLC, oncogenes may be more important drivers of tumour PD‐L1 expression compared to other tumours, like melanoma, in which it seems more influenced by infiltrating immune cells.^69^ Furthermore, as reported by Lyford‐Pike et al, in head and neck squamous cell carcinoma, the expression of PD‐L1 may be driven by both oncogenic and adaptive immune resistance mechanisms in the same lesion.^70^

Therefore, the evaluation of PD‐L1 expression alone as prognostic marker can be misleading, suggesting the need for the integration of other immune markers to obtain a better patient stratification. This action should consider the different phases, which are linked to patients' management. In this view, according to Bigras et al,^71^ the use of small biopsies misclassified up to the 35% of PD‐L1 assessments in advanced NSCLC. The biopsy sample undergoes different processes for the evaluation of PD‐L1 expression. De Meulenaere et al^72^ reported that pathologists can find hurdles in the choice of assay, antibody and cut‐off/score selection of PD‐L1 expression. In this study, authors compared the results of PD‐L1 expression coming from biopsy samples versus resection specimens and a poor agreement emerged. Another study,^73^ on the other hand, showed that the VENTANA PD‐L1 (SP263) assay was characterized by high reproducibility, meanwhile tumour‐infiltrating PD‐L1 immune cells were more variable within and between blocks and across cut‐offs. These data are important for the concept of precise medicine, according also to the evidence that microenvironment has an important role in PD‐L1 expression and tumour behaviour, as showed in other kinds of cancer^74^ and in OSCC.^33^, ^37^, ^40^ According to these statements, future research should focus on the validation and standardization of all steps, from biopsy, IHC assay and tumour microenvironment evaluation for the selection of patients, who can undergo anti‐PD‐L1 therapy.

In order to investigate the influence of specific parameters on the results of this study, we also performed sensitivity analysis for risk of bias, cut‐off values and geography. Summary effect size did not substantially change in sensitivity analyses performed including only studies at low risk of bias, performed in Asia and reporting the same cut‐off value. Results of this study revealed a significant association between PD‐L1 expression and female gender. In fact, in women, higher expression of PD‐L1 seems to be more common as already reported for NSCLC.^75^, ^76^ In these studies, the female subset of patient also corresponds to patients who are more likely to harbour EGFR mutations, suggesting a relationship between PD‐L1 expression and altered EGFR signalling pathway.^77^ A recent meta‐analysis revealed that the magnitude of benefit of patients treated with immunotherapy is sex‐dependent, and in particular, women have lower rates of positive response to the treatment.^78^ However, it is not clear whether such different outcomes are due to the more frequent expression of PD‐L1 in females or to other sex‐related mechanism. Such findings underline the importance of performing future studies aiming to compare sex‐related expression as independent prognostic factor, in order to clarify whether PD‐L1 could be considered a prognostic factor in men but not in women.

Furthermore, as previously mentioned, there is a complex relationship between PD‐L1 expression and the presence and pattern of inflammatory infiltrate. This must be considered in the evaluation of prognostic significance of PD‐L1, because peritumoral inflammatory process seems to be more intense in female patients with OSCC, mainly due to postmenopausal inflammatory state.^19^

Analysis of risk of bias in the included studies revealed deficiencies in some parameters of the REMARKS guidelines. In particular, the authors recorded ambiguity in some of the included studies in the distinction between OS and disease‐specific survival. As it is known, in the calculation of OS, death for any reason is taken into consideration while in disease‐specific survival only deaths for cancer are considered. It is to underline that direct contact of authors helped to clarify such discrepancy for two of the included studies.^23^, ^24^ To note, such meta‐analysis presents some limits, first of all, it relied on published results rather than on individual patients' data. In addition, it presented, for the survival outcomes considered in the meta‐analysis, a very high rate of heterogeneity was detected, that strongly limits the quality of evidences despite the inclusion of an adequate number of studies performed in a good quality manner. Such heterogeneity could reflect the wide variation of PD‐L1 expression in the population that limits its use as prognostic biomarker in clinical practice. It should be stressed that such results are not related to the analysis of PD‐L1 expression as predictor of response to checkpoint inhibitors, such topic should be evaluated in further studies with different design.

## 5 CONCLUSION

High PD‐L1 expression did not correlate with poor prognosis of patients suffering for OSCC. The studies published on the topic showed a significant variation in results. Hence, results from the current available literature limit the use of PD‐L1 expression by immunohistochemistry as prognostic biomarker in clinical practice. Higher levels of PD‐L1 expression are more frequent in females than in males, and such factor should encourage future studies on the sex‐related role of this biomarker.

## CONFLICT OF INTEREST

The authors declare that they have no conflict of interests.
