# Supplementary material for: High PD‐L1 expression in the tumour cells did not correlate with poor prognosis of patients suffering for oral squamous cells carcinoma: A meta‐analysis of the literature
Source: Cell Prolif. 2018 Nov 15;52(2):e12537. doi: 10.1111/cpr.12537 (PMC6495964; doi:10.1111/cpr.12537)
Supplement: Supplementary file 1 [file CPR-52-e12537-s001.docx]

| ***Study Selection*** | ***Reviewer #1*** | ***Reviewer #2*** |
| --- | --- | --- |
| ***Ahn/2017*** | I | I |
| ***Chen/2012*** | E | E |
| ***Chen/2015*** | U | U |
| ***Cho/2011*** | I | I |
| ***Foy/2017*** | U | U |
| ***Fuse/2016*** | E | E |
| ***Hanna/2017*** | I | I |
| ***Hirai/2017*** | U | U |
| ***Jiang/2016*** | E | E |
| ***Katou/2007*** | E | E |
| ***Kogashiwa/2017*** | I | I |
| ***Kubota/2017*** | E | I |
| ***Lanzel/2016*** | E | E |
| ***Lin/2015*** | I | I |
| ***Malaspina/2011*** | E | E |
| ***Maruse/2018*** | E | E |
| ***Mattox/2017*** | E | I |
| ***Oliveira-Costa/2015*** | I | I |
| ***Poropatich/2017*** | E | E |
| ***Ritprajak/2015*** | E | E |
| ***Satgunaseelan/2016*** | U | I |
| ***Stasikowskakanicka/2017*** | E | E |
| ***Straub/2016*** | I | I |
| ***Takahashi/2016*** | E | E |
| ***Troeltzsch/2016*** | U | U |
| ***Weber/2017*** | E | E |
| ***Wu/2017*** | E | E |

**Supplemental Materials:** List of studies to be included in the meta-analysis for the k-agreement calculation.

| **REVIEWER #1** | **REVIEWER #2** | | | | |
| --- | --- | --- | --- | --- | --- |
|  |  |  |  |  |  |
|  |  | **INCLUDE** | **EXCLUDE** | **UNSURE** | **TOTAL** |
|  |  |  |  |  |  |
|  | **INCLUDE** | 7 | 0 | 0 | 7 |
|  |  |  |  |  |  |
|  | **EXCLUDE** | 2 | 13 | 0 | 15 |
|  |  |  |  |  |  |
|  | **UNSURE** | 1 | 0 | 4 | 5 |
|  |  |  |  |  |  |
|  | **TOTAL** | 10 | 13 | 4 | 27 |
|  |  |  |  |  |  |
