# Supplementary material for: High PD‐L1 expression in the tumour cells did not correlate with poor prognosis of patients suffering for oral squamous cells carcinoma: A meta‐analysis of the literature
Source: Cell Prolif. 2018 Nov 15;52(2):e12537. doi: 10.1111/cpr.12537 (PMC6495964; doi:10.1111/cpr.12537)
Supplement: Supplementary file 2 [file CPR-52-e12537-s002.docx]

**Supplemental material:** List of excluded studies and reasons for their exclusion.

| **Study** | **Reasons for exclusion** |
| --- | --- |
| Chen/2012 | Not in human |
| Chen/2015 | PD-L1 expression in necrosis-samples |
| Foy/2017 | Uncompleted Data (no email answer) |
| Fuse/2016 | Not in human |
| Hanna/2017 | Data-characterization on gender and not on PD-L1 expression level |
| Jiang/2016 | Not performed survival analisys |
| Katou/2007 | Not performed survival analisys |
| Kubota/2017 | Survival analisys was not performed according to PD-L1 expression on tumor cells |
| Lanzel/2016 | Not in human |
| Malaspina/2011 | Not performed survival analisys |
| Maruse/2018 | Disease-specific survival analisys |
| Poropatich/2017 | Head and Neck SCC |
| Ritprajak/2015 | Not performed survival analisys |
| Stasikowskakanicka/2017 | Not performed survival analisys |
| Takahashi/2016 | Survival analisys was not performed according to PD-L1 expression on tumor cells |
| Weber/2017 | Not performed survival analisys |
| Wu/2017 | Not performed survival analisys |
