# Supplementary figures and images for: High PD‐L1 expression in the tumour cells did not correlate with poor prognosis of patients suffering for oral squamous cells carcinoma: A meta‐analysis of the literature
Source: Cell Prolif. 2018 Nov 15;52(2):e12537. doi: 10.1111/cpr.12537 (PMC6495964; doi:10.1111/cpr.12537)

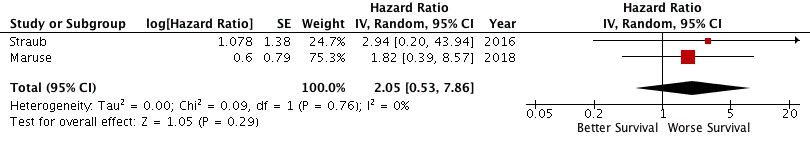

Supplement: Supplementary file 3 [file CPR-52-e12537-s003.png]

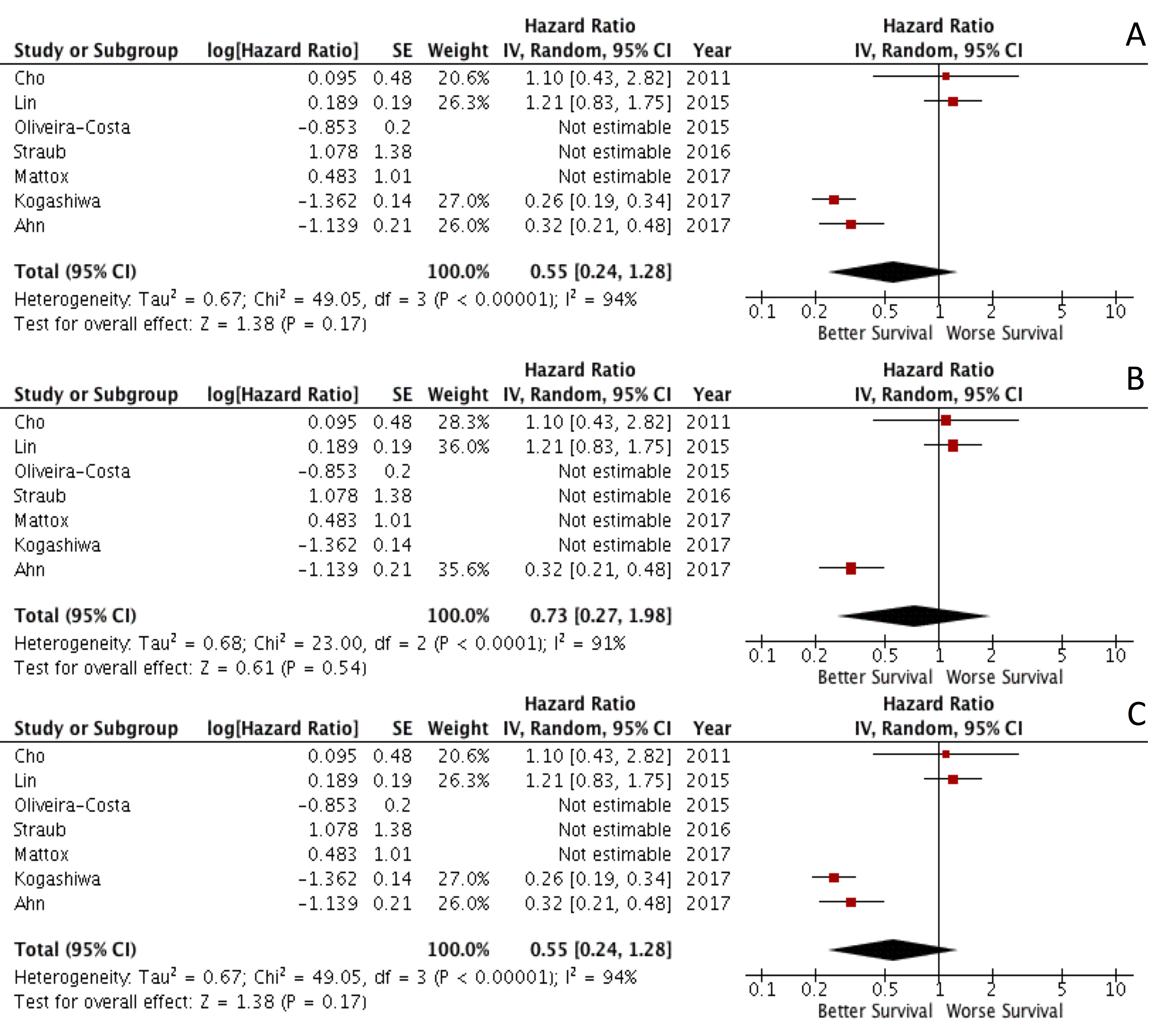

Supplement: Supplementary file 4 [file CPR-52-e12537-s004.png]
